# Corpus Luteum Extract

**Published:** 1912-10

**Authors:** 


Corpus Luteum Extract.—C. F. Burnam, reviewing the function of the internal secretion of the ovaries and the use of the extract, gives his personal experience in experimenting with the extract of the corpus luteum of the pig. When given by the mouth corpus luteum tissue of the sow, even in large doses, has little or no toxic effect on women. It affords a valuable means of controlling the nervous symptoms which occur in so many patients at the time of the natural or artificial menopause, giving relief to most sufferers. It is a valuable remedy in treating patients with insufficient internal ovarian secretion during the menstrual life. This class constitutes a very large number of women. It is an excellent remedy to induce menstruation in young women suffering from functional amenorrhea. Those who are fat, in addition to regaining menstruation, usually, but not always, lose weight. There would seem to be a possibility for the use of the drug in cases of unexplained sterility and repeated abortions. Extensive use should be made of corpora lutea from the cow, sheep, and other animals to determine if these extracts work more successfully than those of the sow. The ideal luteum tissue for any animal is doubtless tissue from its own species, but this can not be obtained for the woman. So far as it goes, the author's work strengthens his conviction that Fraenkel is correct in attributing menstruation in the internal secretion of the corpus luteum. From clinical experiences he is inclined to believe that the corpus luteum possesses different properties due to different chemicals. One of these substances causes hyperemia of the pelvic organs; another relieves nervous symptoms of a toxic character, as at the menopause. It would seem that this product acts as a neutralizer, since even large doses of the luteum cause no disturbance of a toxic nature. On the other hand, the toxic results of intravenous injections of the luteum extracts, as well as the nervous phenomena of menstruation, show that there must also be some toxic material present which is not absorbed from the stomach or intestines. All of these various substances may in the future be separated.—*New York Medical Journal.*

